# A second branchial cleft cyst, a case report

**DOI:** 10.1016/j.ijscr.2023.108429

**Published:** 2023-06-20

**Authors:** Safaa Hadi Abdulsattar Alshihmani

**Affiliations:** Al-kindy Teaching Hospital, Baghdad, Iraq

**Keywords:** Case report, Branchial fistulas, Branchial cysts, General surgery, Second branchial cyst, Bailey-proctor classification

## Abstract

**Introduction & importance:**

Branchial fistulas and cysts are uncommon anomalies of embryonic development that involve soft tissues of the neck. According to Bailey-Proctor classification, second BCCs are classified into four types: Type-I cysts are situated along the anterior border of the sternocleidomastoid muscle beneath the superficial cervical fascia. Type-II ones are the most common and lie just laterally to great vessels beneath enveloping fascia of the neck. Type-III ones pass between internal and external carotid arteries. Type-IV cysts are situated in the pharyngeal mucosal space just deep to the palatine tonsil and medial to great neck vessels, often extending upward towards the skull base. Most second BCCs comprise the first three types, while type-IV cysts are extremely rare.

**Case presentation:**

17 years old male patient from Baghdad/Iraq, single, a student, and living with his family.

**Clinical discussion:**

The patient presented to Al-kindy Teaching Hospital for general surgery consultation due to the presence of a lump in the upper third of the anterior border of the sternocleidomastoid muscle for several years ago, the lump was painless but gradually increase in size with discomfort but without fever, anorexia or weight loss. There were no relieving factors. Regarding the review of systems nothing positive and the history was negative also patient had no past drug history and no psychological illness.

Physical examination of the lump showed a smooth, non-tender, fluctuant cyst located at the upper third of the anterior border of the left sternocleidomastoid muscle about 7 × 4 cm and there were no enlarged lymph nodes. Regarding examination of the other systems there was nothing positive.

Laboratory and radiological investigation showed that the cystic lesion mostly was a branchial cyst, so the operation is done for the patient by complete excision of the cyst with its tract which was located between external and internal carotid vessels. A histopathological study revealed a squamous epithelium-lined cyst with lymphoid infiltration, consistent with a branchial cleft cyst. The patient was discharged without any complication or any evidence of recurrence for 14 months follow-up.

**Conclusion:**

Branchial anomalies remain asymptomatic and can present later in life. They can be misdiagnosed. CT scans and MRI neck are helpful in the diagnosis of the cyst and its anatomical extensions. A proper history and physical examination are required to look for other anomalies like craniofacial syndromes. The treatment of branchial cyst is complete surgical excision to prevent recurrence and removal of these lesions at an earlier stage will increase the quality of life of the patient. In addition, since they are rarely malignant, more successful results will be obtained with early diagnosis and treatment.

## Highlights

• Branchial fistulas and cysts are uncommon anomalies of embryonic development.
• Second BCCs are classified into four types.
• Most second BCCs comprise the first three types, while type-IV cysts are extremely rare.
• Branchial anomalies remain asymptomatic and can present later in life.
• The treatment of branchial cyst is complete surgical excision to prevent recurrence.

## 1 Introduction and importance

Branchial fistulas and cysts are rare embryonic developmental abnormalities affecting the soft tissues of the neck [1]. The six paired branchial arches, that emerge during the fourth week of embryonic life form many of the structures of the head and neck [1]. Among children presenting with cervical swelling, branchial anomalies were present in 17 % of cases. These include branchial cyst, branchial fistula, and branchial sinuses [2].

According to Bailey-Proctor classification, second BCCs are divided into four types: Type-I cysts are located along the anterior border of the sternocleidomastoid muscle beneath the superficial cervical fascia. Type-II ones are the most common and located just laterally to great vessels beneath enveloping fascia of the neck. Type-III ones pass between internal and external carotid arteries. Type-IV cysts are located in the pharyngeal mucosal space just deep to the palatine tonsil and medial to great neck vessels, often extending upward towards the skull base. Most second BCCs comprise the first three types, while type-IV cysts are extremely rare [3].

This work has been reported in line with the SCARE criteria [4].

## 2 Case presentation

17 years old male patient from Baghdad/Iraq, single, student, living with family.

The patient presented to Al-kindy Teaching Hospital for general surgery consultation due to a lump in the upper third of the anterior border of the sternocleidomastoid muscle for several years ago, the lump was painless but progressively enlarged with discomfort but without fever, anorexia or weight loss. There were no relieving factors.

The review of the system was anything but positive, and the medical history was negative. In addition, the patient had no history of drug use or psychiatric illness.

**Fig. 1:**
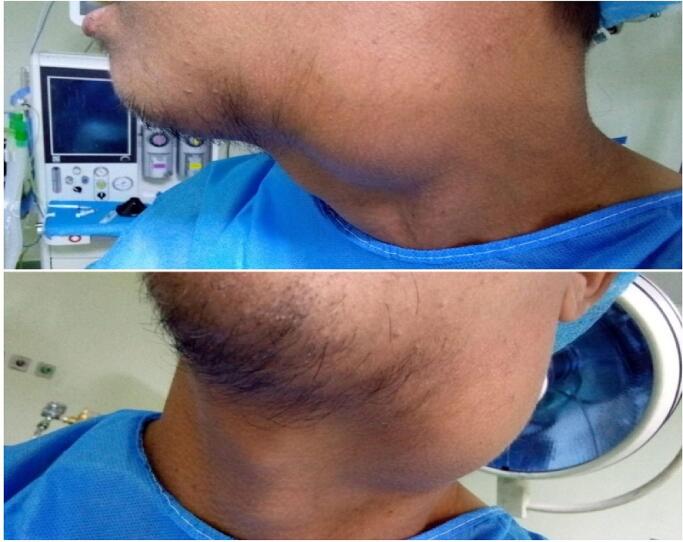
The lump in the upper third of the anterior border of the left sternocleidomastoid muscle.

Physical examination of the lump revealed a smooth, non-tender, fluctuant cyst approximately 7 × 4 cm in the upper third of the anterior border of the left sternocleidomastoid muscle, with no enlarged lymph nodes. There is nothing positive about the research on other systems, Fig. 1.

Laboratory tests were normal, and FNA showed a large number of lymphocytes, foamy histiocytes, and cystic accumulations of lymphoid tissue without malignancy.

With regard to radiology, U/S showed a cystic lesion without evidence of infection, whereas CT scan showed a well-circumscribed hypodense cystic lesion displacing the vascular capsule, independent of the deep parapharyngeal space.

**Fig. 2:**
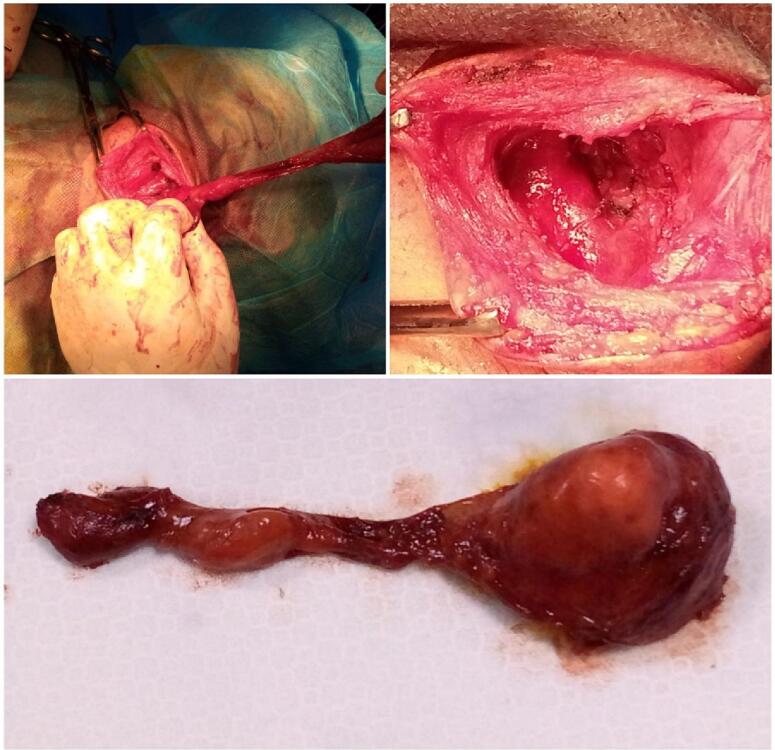
The cyst with its duct located between the external and internal carotid arteries.

**Fig. 3:**
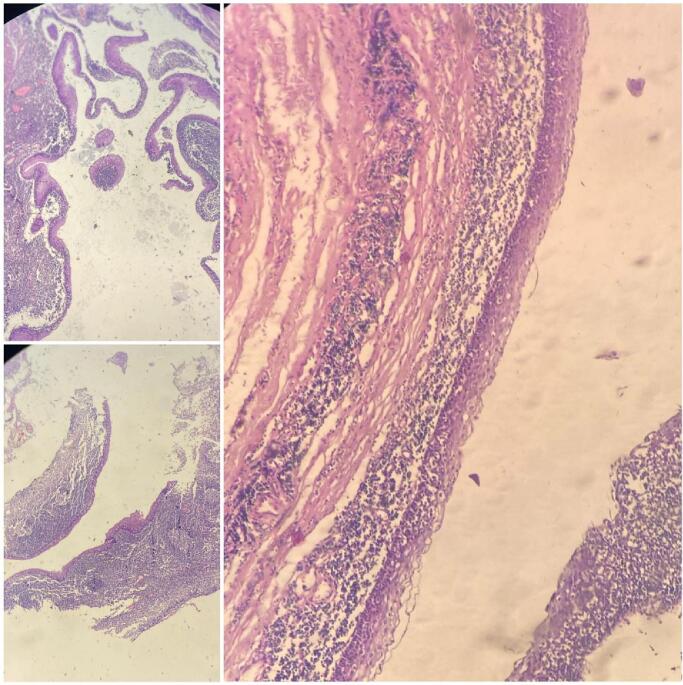
Histopathological examination revealed a squamous-lined cyst with lymphoid infiltration consistent with a branchial cyst.

The operation is performed on the patient under general anesthesia through an elliptical incision over the cyst and complete removal of the cyst with its duct located between the external and internal carotid arteries in order to ligature the end of the duct and close it in layers (Fig. 2). Histopathological examination revealed a squamous-lined cyst with lymphoid infiltration consistent with a branchial cyst (Fig. 3). The patient was discharged with no complications or signs of recurrence during the 14-month follow-up.

## 3 Clinical discussion

Clinically, the remainder of a branchial cleft, which accounts for the majority of branchial cleft anomalies, can present a variety of morphologic patterns, such as a cyst, a sinus, or a fistula. The branchial cleft cyst most commonly occur between the second and fourth decades of life [5]. A branchial sinus or fistula is more common in neonatal period or young age while cyst is rare [5]. Second branchial cleft anomalies are the most common branching anomalies of the apparatus, accounting for about 95 % of cases [6]. First described by Bailey in 1929, the second BCC is a remnant arising from incomplete degeneration of the second branchial arch, as it develops over a long period of time and occurs clinically and progressively later, making diagnosis difficult [7]. According to King's criteria, any cyst that protrudes beyond the midline of the neck and shows lymphoepithelial features should be considered a branchial cyst [8].

The Bailey classification divided anomalies of the second branchial arch into four groups [9]. Type I is the most superficial type, just below the superficial fascia of the neck, in front of the sternocleidomastoid muscle. Type II, the most common type, is found under the cervical fascia medial, anterior, and lateral to the cervical vessels. Type III extends into the pharynx between the internal and external carotid arteries, while the very rare type IV is located lateral to the pharyngeal wall and medial to the carotid artery [9].

The type III patient referred to in this article lies between the internal and external carotid arteries. A history of a positive transillumination reaction and variable edema character to exclude the possibility of cystic hygroma, as well as solid nodules such as tumors of various neck tissues and cervical lymphadenitis and other vascular malformations should be excluded [10]. FNAC provides strong evidence for the initial diagnosis, regardless of whether the lesion is a benign cyst or metastatic carcinoma [11].

The typical ultrasound image of abranchial cyst has been described as a well-defined anechoic cyst, while computed tomography (CT) and magnetic resonance imaging are helpful in evaluating the cyst. Well-circumscribed, homogeneous cystic lesion that may be unilocular or detach in case of secondary infection, as usually seen on CT [11]. The final treatment is surgical removal. However, complicated cysts, such as those with infection or abscess formation, require antibiotic treatment prior to surgery [12].

## 4 Conclusion

Branchial anomalies remain asymptomatic and may appear later in life. They can be misdiagnosed. It is mandatory to confirm the extent of the tract before surgery is considered. Computed tomography and magnetic resonance imaging of the neck are helpful in diagnosing the cyst and its anatomical extensions. Adequate history and physical examination are required to detect other abnormalities such as craniofacial syndromes. Treatment for a branchial cyst is complete surgical removal to prevent recurrence. Early removal of these lesions improves the patient's quality of life. Because they are rarely malignant, early diagnosis and treatment can have better outcomes.

## Ethical approval

The proposal of the study was discussed and approved by scientific & ethical committee in the author teaching hospital. The agreement of health authority in author institution approved before collecting data.

## Consent

Written informed consent was obtained from the patient for publication of this case report and accompanying images. A copy of the written consent is available for review by the Editor-in-Chief of this journal on request.

## Funding

N/A.

## Declaration of competing interest

N/A.
